# UBD‐mediated glycolytic reprogramming promotes M2 macrophage polarization in ovarian cancer immune evasion

**DOI:** 10.1002/ccs3.70034

**Published:** 2025-07-21

**Authors:** Nana Zhang, Fengming Zhao, Hailong Chen, Juli Wang, Haiyan Li

**Affiliations:** ^1^ Obstetrics Department of Shijiazhuang People's Hospital Shijiazhuang China; ^2^ Department of Neurosurgery The Third Hospital of Shijiazhuang Shijiazhuang China; ^3^ Gynecology Department of Shijiazhuang People's Hospital Shijiazhuang China

**Keywords:** glycolytic metabolism, immunotherapy resistance, macrophage polarization, ovarian cancer, tumor microenvironment, ubiquitin D

## Abstract

Ovarian cancer (OC) is one of the most common malignant tumors in women, with immunotherapy resistance (ITR) being a major challenge. Glycolytic metabolic reprogramming has been shown to play a crucial role in the tumor immune microenvironment and immune evasion, yet the underlying mechanisms remain unclear. This study aims to investigate the role of Ubiquitin D (UBD) in OC immunotherapy, particularly its regulation of macrophage polarization through glycolytic metabolism. Using data from the Cancer Genome Atlas and Clinical Proteomic Tumor Analysis Consortium databases, combined with proteomics techniques, we analyzed the expression of UBD in OC tissues and its correlation with key glycolytic enzymes. Through lentiviral‐mediated gene manipulation and in vivo mouse models, we evaluated the effects of UBD on macrophage polarization, glycolytic metabolism, and immunotherapy. The results indicate that UBD promotes M2 macrophage polarization through glycolytic reprogramming, enhancing immune evasion and ITR in OC. Inhibiting UBD or targeting glycolytic pathways may provide new strategies for improving OC immunotherapy.

## Graphical abstract

Multimodal data analysis reveals the molecular mechanism by which UBD‐mediated glycolytic metabolic reprogramming regulates macrophage polarization and influences immune therapy resistance in OC.

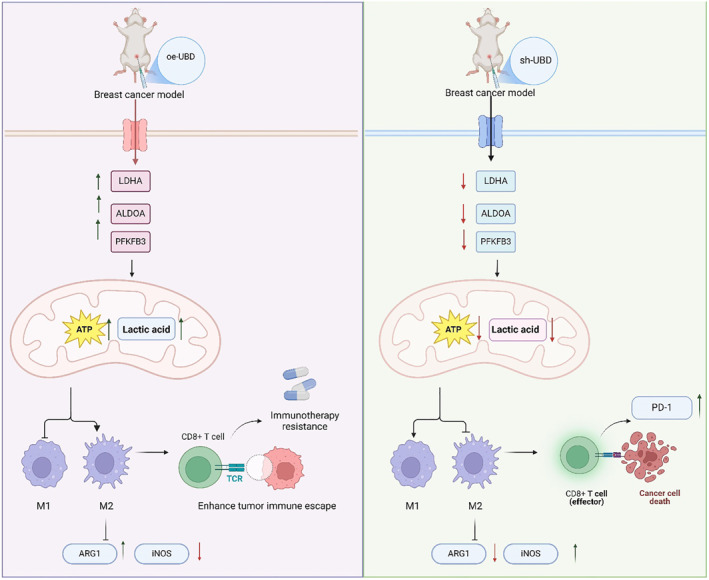

## 1 INTRODUCTION

Ovarian cancer (OC) is one of the most common malignant tumors of the female reproductive system. Despite advancements in medical technology that have improved early diagnosis and treatment, most patients are diagnosed at advanced stages, leading to a relatively low 5‐year survival rate.^1^ Although chemotherapy, targeted therapy, and immunotherapy have made significant progress, relapse and drug resistance remain major challenges for the majority of OC patients.^2^, ^3^ Immune evasion has been identified as a key mechanism in OC progression, particularly through the modulation of immune cell function within the tumor microenvironment (TME). Macrophages, essential immune cells in the tumor immune microenvironment, regulate immune responses by polarizing into different subtypes. In the TME, macrophages typically adopt an M2 polarization, which promotes immune evasion, tumor progression, and immune resistance.^4^, ^5^, ^6^ Studies have shown that M2 macrophages secrete immunosuppressive factors, such as IL‐10 and TGF‐β, to suppress CD8^+^ T cell function, thereby facilitating immune evasion of tumor cells.^7^, ^8^, ^9^

Glycolytic metabolism is a reprogrammed metabolic pathway that tumor cells adapt to support rapid proliferation.^10^, ^11^, ^12^ Unlike normal cells, tumor cells preferentially utilize glycolysis for energy production even under normoxic conditions, a phenomenon known as the “Warburg effect”.^13^, ^14^ Not only tumor cells but also immune cells in the TME, particularly macrophages, exhibit metabolic changes that significantly influence immune responses. Research studies have shown that M2 macrophages, regulated by glycolytic pathways, enhance their immunosuppressive functions through elevated lactate production and metabolic adaptation to hypoxic conditions.^15^, ^16^ Glycolysis not only provides energy for macrophages but also promotes tumor growth and metastasis by modulating the acidic microenvironment and cytokine secretion.^17^, ^18^ Consequently, glycolytic metabolism has become an increasingly important focus of research as a key mechanism in tumor immune evasion.

Ubiquitin D (UBD) is a ubiquitin‐like protein that plays a crucial role in regulating the cell cycle, chromosomal instability, apoptosis, and immune responses. Abnormal expression of UBD is frequently associated with tumorigenesis and progression in various cancers.^19^, ^20^ Increasing evidence suggests that UBD plays a significant role in tumorigenesis and also influences tumor immune evasion by regulating metabolic pathways.^21^ UBD contributes to immune evasion in tumors by modulating glycolytic metabolism and promoting the M2 polarization of macrophages. In several malignancies, including OC, UBD expression is upregulated and closely associated with the upregulation of glycolysis‐related genes such as *LDHA* and *ALDOA*. The elevated expression of these key enzymes enhances M2 macrophage polarization, thereby facilitating immune evasion in tumors.^22^, ^23^ Additionally, studies indicate that UBD regulates key enzymes in glycolysis, altering the metabolic state of macrophages and driving them toward an immunosuppressive phenotype.^24^ Thus, as a regulator of glycolytic metabolism, UBD plays a crucial role in the immune evasion mechanisms of OC.

Although previous studies have established the critical role of macrophage polarization in tumor immune responses, the specific mechanisms by which UBD regulates macrophage polarization through metabolic pathways to influence immune evasion remain unclear. This study aims to elucidate the role of UBD in OC immune evasion and resistance to immunotherapy, particularly focusing on its modulation of macrophage polarization through glycolytic metabolic reprogramming. Immune evasion in OC is closely linked to the metabolic reprogramming of tumor cells, with glycolysis playing a crucial role in the tumor immune microenvironment. Thus, the novelty of this study lies in its proposal that UBD may regulate macrophage polarization by modulating glycolytic reprogramming, thereby impacting immune evasion and resistance to immunotherapy in OC. By investigating the relationship between UBD and key enzymes in the glycolytic pathway, this research study aims to provide new theoretical insights into OC immunotherapy and explore potential clinical intervention strategies.

## 2 MATERIALS AND METHODS

### 2.1 Ethical statement

This study strictly adheres to ethical guidelines and regulations governing animal experiments. All experimental procedures were approved by the Institutional Animal Care and Use Committee (IACUC) (Ethical Approval Number: No. 120241 (005)). The animals were housed and cared for in accordance with humane principles, and efforts were made to minimize any potential pain or distress during the experiments.

### 2.2 Public data download

To analyze the expression of UBD in OC, transcriptomic data and corresponding clinical information from OC patients were downloaded from the Cancer Genome Atlas (TCGA) database (https://portal.gdc.cancer.gov/). The downloaded dataset includes RNA‐Seq data (RSEM values) from hundreds of OC patients and normal ovarian tissues, with data formatted as FPKM (fragments per kilobase of exon per million reads mapped). The RNA‐Seq data were normalized and subsequently used for differential expression analysis, survival analysis, and other investigations. The clinical data for the samples include patient survival time, age, and pathological stage.

To explore the association between UBD and glycolytic pathways, proteomic data for OC patients were downloaded from the Clinical Proteomic Tumor Analysis Consortium (CPTAC) (https://proteomics.cancer.gov/data‐portal) and PRIDE (https://www.ebi.ac.uk/pride/archive) databases. The data included multiple OC samples and their corresponding normal tissue samples, formatted as label‐free quantification (LFQ) values generated by MaxQuant. Data processing was performed using Perseus software (v1.6.15.0, Max Planck Institute of Biochemistry, Germany), which involved filtering out proteins with more than 30% missing values, performing log2 transformation, and normalizing protein expression levels to ensure suitability for differential expression and network analysis.

### 2.3 Differential expression analysis

To evaluate the differential expression of the UBD gene between OC tissues and normal tissues, we employed the DESeq2 package (v1.32.0) in R. First, we imported the normalized RNA‐Seq data and constructed a DESeqDataSet object. Differential expression analysis was then performed to compare the expression levels of the UBD gene in OC tissues and normal tissues. Multiple testing correction was conducted using the Benjamini–Hochberg method, with a significance threshold set at *p* < 0.05 and |log2FoldChange| > 1. Genes with significant differential expression were identified. Data visualization was carried out using the ggplot2 package (v3.3.5) in R, generating volcano plots and heatmaps to visually display the distribution of UBD and other significantly differentially expressed genes.

### 2.4 Co‐expression analysis

We conducted a weighted gene co‐expression network analysis (WGCNA) to investigate the co‐expression relationship between the UBD gene and glycolysis‐related metabolic genes. Using the WGCNA package (v1.70) in R, we first filtered the RNA‐Seq data to remove low‐expression genes. Next, a gene co‐expression network was constructed based on Spearman's correlation coefficient, focusing on key glycolysis‐related genes (ALDOA, PFKP, PGK1, and LDHA) and evaluating their correlation with UBD.

### 2.5 Survival analysis

To assess the relationship between UBD gene expression and prognosis in OC patients, we performed Kaplan–Meier survival analysis using the survival (v3.2‐11) and survminer (v0.4.9) packages in R. Patients were stratified into high and low expression groups based on the median level of UBD expression. Log‐rank tests were used to compare survival curves between the two groups, determining the association between UBD expression and overall survival.

### 2.6 Immune infiltration analysis

To investigate the relationship between the UBD gene and immune cell infiltration, we employed the CIBERSORT tool to analyze the immune cell composition in OC samples. After processing the RNA‐Seq data with CIBERSORT, we determined the proportion of each immune cell type within the TME. Furthermore, we explored the correlation between the infiltration of M2 macrophages and the expression levels of the UBD gene. The correlation scatter plot was generated using the ggplot2 package (v3.3.5) in R, and the correlation coefficient was calculated to assess the relationship between UBD expression and immune infiltration.

### 2.7 Differential protein expression analysis

Differential protein expression was analyzed using the limma package (v3.48.3) in R. The samples were divided into high and low UBD expression groups, and the expression differences of glycolysis‐related proteins between the two groups were compared. Multiple testing correction was performed using the Benjamini–Hochberg method, with a significance threshold set at a corrected *p*‐value <0.05. Differential proteins were selected based on a |log2FoldChange| > 1. To better visualize the results, a volcano plot of differentially expressed proteins was generated using the ggplot2 package (v3.3.5), and a heatmap illustrating the expression of significantly altered proteins was created using the pheatmap package (v1.0.12).

### 2.8 Proteomics network analysis

To construct an interaction network between UBD and glycolysis‐related proteins, we utilized the STRING database (https://string‐db.org/) and selected proteins with an interaction score greater than 0.4. The resulting protein–protein interaction network was visualized using Cytoscape software (v3.8.2).

### 2.9 Co‐expression analysis

Co‐expression analysis was performed using the WGCNA package (v1.70‐3) in R. First, low‐expressed and highly missing proteins were filtered out to retain a high‐quality dataset. The Spearman correlation coefficient was calculated to analyze the co‐expression relationships between UBD and glycolysis‐related proteins (LDHA, SLC2A1, PKM, PGK1, and ALDOA). A co‐expression heatmap was generated using the pheatmap package (v1.0.12) to visualize the co‐expression patterns between UBD and glycolysis‐related proteins.

### 2.10 Cell culture

RAW264.7 macrophage cells (CL‐0190, Wuhan Pricella Biotechnology Co., Ltd., Wuhan) and the human OC cell line A2780 (CBP60283, COBIOER, Nanjing) were used as model cells. RAW264.7 cells were cultured in high‐glucose DMEM (11,965‐092, Gibco, USA) supplemented with 10% Fetal Bovine Serum (FBS) (FBS, 10,099‐141, Gibco, USA), 1% penicillin‐streptomycin (15,140‐122, Gibco, USA), and 1% L‐glutamine (25,030‐081, Gibco, USA). A2780 cells were cultured in RPMI 1640 (11,875‐119, Gibco, USA) supplemented with 10% FBS, 1% penicillin‐streptomycin, and 1% L‐glutamine. All cells were maintained in a 37°C incubator with 5% CO_2_ (Heracell VIOS 160i, Thermo Fisher Scientific, USA), with medium changes every 2–3 days and subculturing performed at a 1:4 ratio. Prior to experimentation, cells were treated when they reached 80%–90% confluence.^25^, ^26^

### 2.11 Lentiviral‐mediated cell transfection

To investigate the role of UBD in macrophage polarization, we first employed lentiviral‐mediated gene knockdown to reduce UBD expression in macrophages. Specifically, a lentiviral vector containing UBD‐specific shRNA was obtained from Targeting Vector Core (Sigma‐Aldrich, USA), and lentiviral particles were produced in HEK293T cells (ATCC, USA). These viral particles were then transfected into RAW264.7 macrophage cells, and after 72 h, stable knockdown cell lines were selected using puromycin (4 μg/mL, A1113803, Gibco, USA).

Subsequently, we constructed a lentiviral vector for UBD overexpression. The full‐length UBD complementary DNA (cDNA) was obtained from NCBI and cloned into the pLVX‐Puro lentiviral expression vector (Clontech, USA). The vector and UBD cDNA were digested with EcoRI and NotI and ligated to form a recombinant plasmid. This plasmid was co‐transfected with packaging plasmid psPAX2 and envelope plasmid pMD2.G (Addgene, USA) into HEK293T cells to produce lentiviral particles. After 48 h, the viral supernatant was collected and filtered through a 0.45 μm membrane. The lentiviral particles were then used to infect RAW264.7 macrophage cells, and after 72 h, stable overexpression cell lines were selected using puromycin (4 μg/mL). Both knockdown and overexpression efficiencies were validated by reverse transcription quantitative polymerase chain reaction (RT‐qPCR) and Western blot.

The cell transfection groups were as follows: (1) sh‐NC group: cells transfected with a negative control lentiviral vector for gene knockdown; (2) sh‐UBD1# group: cells transfected with a sh‐UBD lentiviral vector for UBD knockdown; (3) sh‐UBD2# group: macrophages transfected with the second shRNA construct targeting UBD; (4) oe‐NC group: cells transfected with a negative control lentiviral vector for overexpression; and (5) oe‐UBD group: cells transfected with an oe‐UBD lentiviral vector for UBD overexpression. After 48 h of transfection, RT‐qPCR was performed to assess mRNA levels and confirm the knockdown and overexpression efficiency. All plasmids used in the study were designed and synthesized by Guangzhou RiboBio Co., Ltd.

### 2.12 RT‐qPCR

To assess the impact of UBD knockdown on M2 and M1 macrophage polarization markers, RT‐qPCR was performed to measure the mRNA expression levels of ARG1 and inducible nitric oxide synthase (iNOS). The primers were designed and provided by Shanghai General Biological Technology Co., Ltd., with the sequences detailed in Table S1. Total RNA was extracted using TRIzol reagent (15596018CN, Invitrogen, USA), and RNA concentration was quantified using NanoDrop 2000 (Thermo Fisher Scientific, USA). cDNA was synthesized using High‐Capacity cDNA Reverse Transcription Kit (Applied Biosystems, USA). RT‐qPCR was conducted using SYBR Green Master Mix (Applied Biosystems, USA) on the StepOnePlus Real‐Time PCR System (Applied Biosystems, USA), with Glyceraldehyde‐3‐Phosphate Dehydrogenase (GAPDH) as the internal reference gene.

### 2.13 Western blot analysis

Proteins were extracted from cell lysates using radio‐immunoprecipitation assay (RIPA) buffer (Thermo Fisher Scientific, USA) and quantified with the Bicinchoninic Acid (BCA) Protein Assay Kit (Thermo Fisher Scientific, USA). After separation by sodium dodecyl sulfate polyacrylamide gel electrophoresis (SDS‐PAGE), proteins were transferred to a polyvinylidene fluoride (PVDF) membrane (Millipore, USA). Primary antibodies against ARG1 (ab203490, 1:1000, Abcam, UK), iNOS (ab178945, 1:1000, Abcam, UK), HK‐2 (ab227198, 1:1000, Abcam, UK), PFKFB3 (ab181861, 1:1000, Abcam, UK), UBD (HY‐P80364, 1:1000, MCE, USA), and GAPDH (ab8248, 1:5000, Abcam, UK) were used for incubation. Subsequently, horseradish peroxidase (HRP)‐conjugated secondary antibody (sc‐2004, 1:5000, Santa Cruz Biotechnology, USA) was applied. Protein bands were detected using an enhanced chemiluminescence (ECL) substrate (Thermo Fisher Scientific, USA).

### 2.14 Immunofluorescence staining

Cells fixed on glass slides were treated with 4% paraformaldehyde (Sigma‐Aldrich, USA) for fixation and subsequently permeabilized with 0.3% Triton X‐100 (Sigma‐Aldrich, USA). Primary antibodies against ARG1 (ab203490, 1:100, Abcam, UK) and iNOS (ab178945, 1:500, Abcam, UK) were applied, followed by incubation with Alexa Fluor 488‐conjugated secondary antibody (ab150077, 1:100, Abcam, UK). Fluorescent signals were observed using a confocal microscope (Leica Microsystems, Germany).

### 2.15 Glucose uptake measurement

To assess the impact of UBD knockdown on macrophage glycolysis, glucose uptake was measured using a glucose uptake assay kit (BioVision, USA). The treated cells were incubated with glucose uptake buffer, and a 2‐NBDG glucose probe (N13195, Thermo Fisher Scientific, USA) was added. After incubation, the cells were washed with Phosphate‐Buffered Saline (PBS) and analyzed using a flow cytometer (BD Biosciences, USA) to measure the fluorescence intensity of 2‐NBDG, which reflects the glucose uptake level.

### 2.16 Co‐culture of macrophages and OC cells

To assess how UBD‐mediated glycolytic reprogramming influences the proliferation, migration, invasion, and immunotherapy sensitivity of OC cells, a co‐culture system was established between macrophages and OC cells. The OC cell line A2780 was used, with the same culture conditions as those for macrophages. In the co‐culture experiments, RAW264.7 macrophages with UBD knockdown or overexpression were seeded in the upper chamber of a Transwell (0.4 μm pore size, Corning, USA), with A2780 OC cells placed in the lower chamber. Co‐cultures were incubated for 24, 48, and 72 h, after which OC cells were collected for further analysis.

To evaluate the impact of glycolytic metabolic regulation on OC ITR, treated macrophages were co‐cultured with A2780 OC cells in the presence of PD‐L1 antibody (10 μg/mL, BioLegend, USA). After 48 h of incubation, cell viability was assessed by Trypan Blue exclusion assay.

### 2.17 CCK‐8 cell proliferation assay

To assess the impact of UBD‐regulated macrophages on OC cell proliferation, a CCK‐8 assay (CK04, Dojindo, Japan) was performed. After co‐culturing for 24, 48, and 72 h, OC cells were harvested from the bottom layer and transferred to a 96‐well plate. Ten microliters of CCK‐8 solution were added to each well, followed by incubation for 2 h. Absorbance was measured at 450 nm using a microplate reader (Bio‐Rad, USA), and the proliferation inhibition or promotion rate for each experimental group was calculated relative to the control group.

### 2.18 Transwell migration and invasion assay

To assess the impact of UBD knockdown or overexpression in macrophages on the migration and invasion of OC cells, Transwell chambers (8 μm pore size, Corning, USA) were used for migration and invasion assays. OC cells were seeded in the upper chamber, with no matrix gel added for the migration assay, and a layer of matrix gel (BD Biosciences, USA) was applied in the upper chamber for the invasion assay. After 24 h of incubation, cells on the bottom of the chambers were fixed with methanol and stained with 0.1% crystal violet (Sigma‐Aldrich, USA). The number of migrating or invading cells was counted and analyzed under a microscope.

### 2.19 PD‐L1 antibody treatment

To investigate the impact of UBD‐mediated glycolytic metabolism on the immune therapy sensitivity of OC cells, a PD‐L1 antibody (0.2 μg/mL, BioLegend, USA) was added to the co‐culture system. After 48 h of incubation, cell viability was assessed using the Trypan Blue exclusion assay. An equal volume of 0.4% Trypan Blue (Thermo Fisher Scientific, USA) was mixed with the cell suspension, and the number of viable and total cells was counted using the Countess II FL Automated Cell Counter (Thermo Fisher Scientific, USA). The cell viability was then calculated.

### 2.20 Glycolysis metabolic regulation experiment

To investigate the role of glycolytic metabolism in UBD‐mediated macrophage polarization, experiments were conducted using the glycolysis inhibitor 2‐DG and the glycolysis activator PFKFB3 agonist. RAW264.7 cells were divided into a UBD overexpression group, a UBD knockdown group, and their respective control groups (oe‐NC and sh‐NC). In the UBD overexpression group, 2‐DG (10 mM, Sigma‐Aldrich, USA) was added to inhibit glycolysis. In the UBD knockdown group, a PFKFB3 agonist (10 μM, Selleckchem, USA) was applied to enhance glycolysis. All treatments were administered for 24 h.

### 2.21 OC mouse model construction

C57BL/6 female mice (6–8 weeks old, purchased from Charles River Laboratories, USA) were used for the OC mouse model construction. ID8 OC cells (iCell‐m064, Cellverse Bioscience Technology Co., Ltd.) were injected intraperitoneally into the left abdomen of the mice at a concentration of 1 × 10^6^ cells in 100 μL PBS.^27^ RAW264.7 macrophage cells were used to prepare UBD knockdown (sh‐UBD), UBD overexpression (oe‐UBD), and corresponding control groups (sh‐NC and oe‐NC). After tumor implantation, treated macrophages (1 × 10^6^ cells/100 μL PBS) were injected locally into the tumor sites of the mice. Each group received one injection per week for a total of 3 weeks. For the PD‐L1 antibody treatment group, PD‐L1 antibody (250 μg, BioLegend, USA) was injected simultaneously at the tumor site immediately after macrophage injection, followed by weekly injections for 3 weeks.^28^, ^29^, ^30^, ^31^ At the end of the experiment, the mice were euthanized, and the tumor tissues were excised, weighed, and fixed.^32^

### 2.22 Flow cytometry analysis

To analyze immune cell infiltration in the TME, single‐cell suspensions were prepared from mouse tumor tissues. The tumor tissues were homogenized and filtered through a 70 μm cell strainer (BD Biosciences, USA), followed by red blood cell (RBC) lysis using RBC lysis buffer (Thermo Fisher Scientific, USA). Flow cytometry analysis was performed using PE Rat Anti‐Mouse CD8a (553,032, BD Biosciences, USA) and FITC Hamster Anti‐Mouse CD3e (553,062, BD Biosciences, USA). CD8^+^ T cell populations were detected using a BD FACSCanto II flow cytometer (BD Biosciences, USA), and the data were analyzed using FlowJo software (v10.7.1, BD Biosciences, USA).

### 2.23 Lactate and ATP levels measurement

Cell samples were extracted from the tumor tissues of each mouse group, and lactate and ATP levels were measured using the Lactate Assay Kit (K607‐100, BioVision, USA) and ATP Assay Kit (A22066, Thermo Fisher Scientific, USA), respectively. The measurements were conducted following the standard protocol provided in the kit instructions, and the results were read using a microplate reader (Bio‐Rad, USA).

### 2.24 Immunohistochemical analysis

To analyze macrophage polarization, immunohistochemical staining was performed on fixed tumor tissue sections. Primary antibodies against ARG1 (ab203490, 1:500, Abcam, UK) and iNOS (ab283655, 1:500, Abcam, UK) were used for labeling, followed by incubation with HRP‐conjugated secondary antibody (1:200, Santa Cruz Biotechnology, USA). Staining was developed using the DAB substrate kit (P0202, Beyotime). After staining, images were captured using a microscope (Leica Microsystems, Germany) and quantitatively analyzed with ImageJ software (v1.53e) to determine the proportion of ARG1‐and iNOS‐positive cells.

### 2.25 Statistical analysis

All experimental data were analyzed using GraphPad Prism 9.0 software (GraphPad Software, USA). Data are expressed as the mean ± standard deviation (Mean ± SD). The normality of the data was first assessed using the Shapiro–Wilk test. For normally distributed data, comparisons between two groups were performed using the independent samples *t*‐test, while multiple group comparisons were made using analysis of variance (ANOVA) followed by Tukey's post‐hoc test. For non‐normally distributed data, nonparametric tests, such as the Mann–Whitney *U* test or the Kruskal–Wallis test, were applied. Data involving time‐point changes were analyzed using repeated measures ANOVA. Correlation analysis was conducted using Pearson or Spearman methods, depending on data distribution. A significance level of *p* < 0.05 was considered statistically significant. Graphs are presented as bar or line charts, with error bars indicating the variability of the data.

## 3 RESULTS

### 3.1 UBD regulates macrophage metabolic reprogramming in OC

**FIGURE 1:**
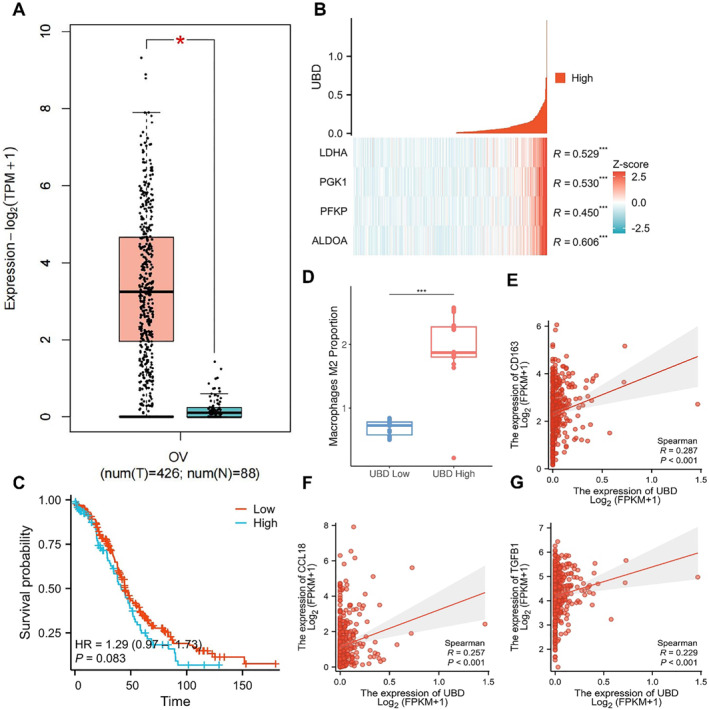
Transcriptomic Analysis Reveals the Expression of UBD in OC and Its Association with Glycolytic Metabolism‐Related Genes. (A) Expression levels of UBD in OC and normal ovarian tissues; (B) Co‐expression analysis of UBD with key glycolytic genes; (C) Kaplan‐Meier survival curve analysis comparing survival rates between high and low UBD expression groups in OC patients; (D) CIBERSORT analysis revealing the correlation between high UBD expression and M2 macrophage infiltration in OC tissues; (E–G) Co‐expression analysis of UBD with M2 macrophage markers (CD163, CCL18, and TGFB1). OC, ovarian cancer; UBD, Ubiquitin D.

In this study, we aim to explore the functional role of UBD in OC and its involvement in metabolic reprogramming and immune microenvironment regulation. First, we examined the differential expression of UBD in OC tissues compared to normal ovarian tissues to determine its potential role in tumorigenesis and progression. Analysis of transcriptomic data from OC patients in the TCGA database revealed that UBD expression was significantly higher in OC tissues than in normal ovarian tissues (Figure 1A). Compared to normal ovarian tissue, the relative expression level of UBD in OC tissue (*n* = 426) was upregulated by approximately 2.5‐fold. This significant difference was observed across multiple subtypes of OC, further suggesting that UBD may play a key role in the development of OC.

To further investigate the mechanism of UBD's action, we focused on its regulatory role in OC cell metabolism, particularly its relationship with glycolysis. Glycolysis, a critical metabolic pathway in tumor cells, is closely associated with tumor growth.^20^, ^33^ Co‐expression analysis of key genes in the glycolysis (Figure 1B) revealed a significant positive correlation between UBD and several glycolysis‐related genes, including ALDOA, PFKP, PGK1, and LDHA. These findings suggest that UBD may promote metabolic reprogramming in OC cells by regulating these glycolysis genes. Specifically, the co‐expression coefficient between UBD and ALDOA was 0.606, indicating that ALDOA's role in glycolysis may be regulated by UBD. The co‐expression coefficient between UBD and PFKP was 0.450, highlighting UBD's potential role in regulating the rate‐limiting enzymes of glycolysis. The co‐expression coefficients between UBD and PGK1 and LDHA were 0.530 and 0.529, respectively, suggesting that these enzymes play a key role in the downstream steps of glycolysis. Based on these results, we hypothesize that UBD may facilitate cancer cell metabolic reprogramming by modulating key steps of glycolysis, thereby providing energy support for tumor cell growth and immune evasion.

Considering the role of UBD in metabolic pathways, we further explored whether its high expression is associated with the prognosis of OC patients. Kaplan–Meier survival analysis revealed a significant correlation between high UBD expression and poor prognosis in OC patients (Figure 1C). The survival analysis indicated that patients with high UBD expression exhibited a declining survival rate over time, particularly after 100 months of follow‐up, where their survival probability was significantly lower than that of patients with low UBD expression. Although there was no significant survival difference in the early stages, the long‐term survival rate was notably worse in the high UBD expression group, suggesting that UBD may serve as a potential negative prognostic marker for long‐term survival in OC patients.

We further examined the role of UBD in the TME, particularly its effect on immune cell infiltration, using CIBERSORT analysis. The results demonstrated a significant association between high UBD expression and the infiltration of M2 macrophages in the TME (Figure 1D). Specifically, OC patients with high UBD expression showed a 1.6‐fold increase in the proportion of M2 macrophages in their tumor tissue compared to those with low UBD expression. This suggests that UBD may promote immune evasion in OC by regulating the accumulation of M2 macrophages in the TME. Further correlation analysis revealed a significant positive correlation between UBD and M2 macrophage markers, such as CD163, CCL18, and TGFB1 (Figure 1E–G).

These findings indicate that UBD may influence immunosuppressive TME by modulating the polarization of M2 macrophages, thereby contributing to the development of immune therapy resistance.

### 3.2 Proteomics analysis reveals a strong correlation between UBD overexpression and upregulation of key proteins in the OC glycolytic pathway, as well as activation of the metabolic network

**FIGURE 2:**
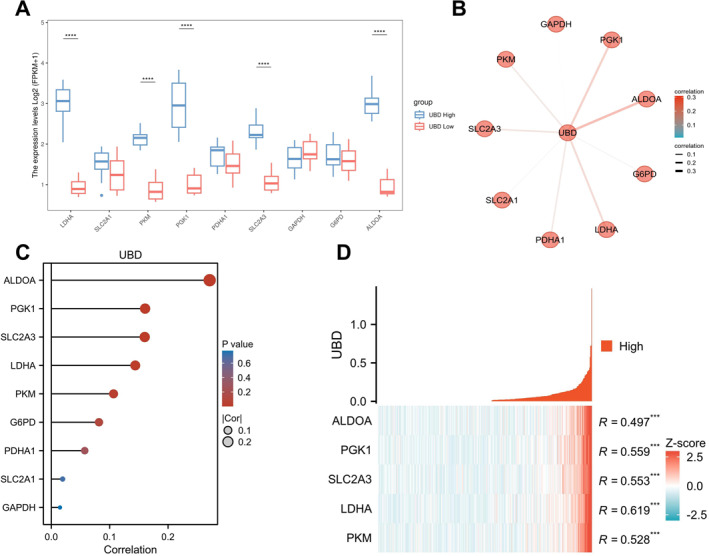
Correlation of UBD and Glycolysis‐related Proteins in ovarian cancer Based on Proteomics Analysis. (A) Expression differences of key glycolysis proteins between high and low UBD expression groups; (B) Proteomics network analysis showing the activation of glycolysis in high UBD expression samples; (C) Correlation analysis between UBD expression levels and glycolysis‐related proteins; (D) Co‐expression analysis of UBD with glycolysis‐related proteins. UBD, Ubiquitin D.

After identifying the potential link between UBD and the glycolytic pathway, we further validated this relationship using proteomics data from the CPTAC public database. Our aim was to determine whether UBD regulates the metabolic activity of OC cells by affecting the expression of key glycolytic enzymes. The analysis showed that in OC tissues with high UBD expression, the levels of key glycolytic enzymes, including LDHA, SLC2A1, PKM, PGK1, and ALDOA, were significantly higher compared to those with low UBD expression (Figure 2A). Specifically, the expression of LDHA was approximately doubled in the UBD high‐expression group, suggesting that UBD may enhance the metabolic activity of cancer cells by promoting lactate production at the end of the glycolytic pathway. Additionally, the expression of SLC2A3, a key glucose transporter in glycolysis, was markedly increased, indicating that UBD overexpression may facilitate glycolytic metabolism by enhancing glucose uptake in cancer cells. Furthermore, the expression of downstream glycolytic enzymes such as PKM and PGK1 was also significantly upregulated, further supporting the notion that UBD regulates glycolysis through these critical enzymes. These findings suggest that UBD overexpression in OC promotes the expression of key glycolytic enzymes, thereby enhancing the cancer cells' energy metabolism and providing metabolic support for tumor growth and immune evasion.

To further investigate the role of UBD in regulating glycolytic metabolism, we conducted a proteomic network analysis focusing on protein interactions within the glycolysis (Figure 2B). We found that SLC2A3, a key transporter in the glycolysis, showed a significant positive correlation with UBD in the network, suggesting that UBD may enhance glucose uptake by upregulating the expression of these transporters, thereby providing sufficient energy for tumor cells. Moreover, the activation of key enzymes such as PKM and LDHA indicates that UBD may accelerate tumor cell energy metabolism by promoting lactate production in the downstream steps of glycolysis. Additionally, PGK1 and ALDOA were also significantly correlated with UBD. PGK1, which generates ATP during glycolysis, may facilitate ATP production through its upregulation, thus meeting the energy demands of cancer cells. The upregulation of ALDOA may promote the accumulation of intermediate metabolites, thereby enhancing overall glycolytic activity. These results suggest that UBD may directly influence the glycolytic pathway by upregulating the expression of these enzymes, thereby regulating the metabolic state and therapy resistance in OC.

To further validate the co‐expression relationship between UBD and glycolysis‐related proteins, we conducted a correlation analysis (Figure 2C). The results revealed a strong positive correlation between UBD and proteins such as ALDOA, SLC2A3, PKM, and LDHA, suggesting that UBD may promote glucose uptake and the activation of metabolic pathways in OC cells by upregulating glycolytic enzymes. Co‐expression analysis further demonstrated a significant positive correlation between UBD and proteins, including PKM, LDHA, SLC2A3, PGK1, and ALDOA (Figure 2D). High UBD expression may facilitate metabolic reprogramming in cancer cells by regulating the expression of these glycolysis‐related proteins, thereby enhancing the metabolic activity and therapeutic resistance of OC. These findings suggest that UBD may serve as a potential therapeutic target, and its inhibition could reduce OC cell dependence on glycolysis, thereby improving the response to existing treatments.

### 3.3 UBD knockdown suppresses M2 macrophage polarization and significantly reduces glycolytic activity and energy metabolism

**FIGURE 3:**
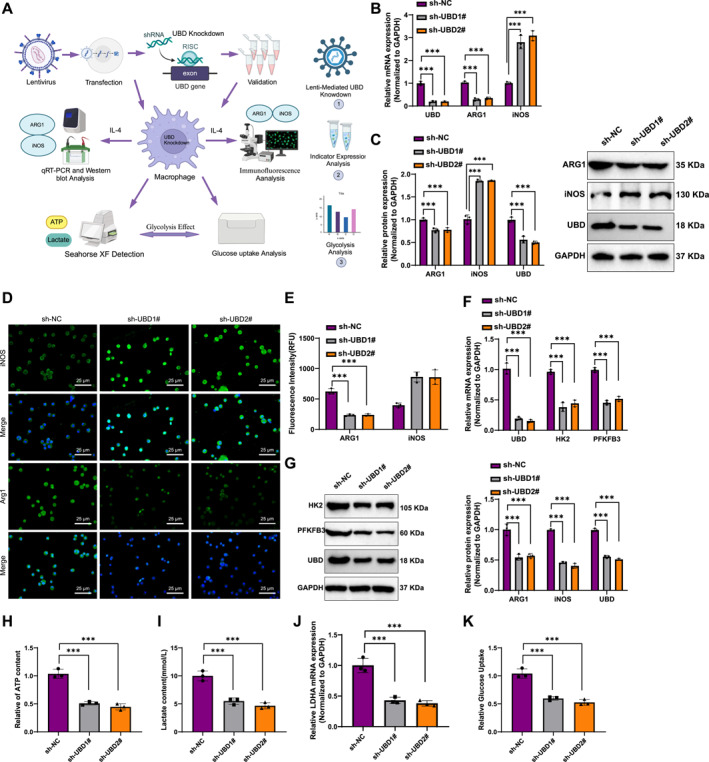
In Vitro Cell Experiments Validating the Effect of UBD on Macrophage Polarization and Glycolytic Metabolism. (A) Experimental workflow for UBD gene knockdown in macrophages mediated by lentivirus; (B) reverse transcription quantitative polymerase chain reaction analysis of mRNA expression levels of M2 macrophage marker ARG1 and M1 macrophage marker iNOS in macrophages; (C) Western blot analysis of protein expression levels of ARG1 and iNOS in macrophages; (D, E) Immunofluorescence staining of ARG1 and iNOS in macrophages to observe changes in fluorescence signals; (F, G) RT‐qPCR and Western blot analysis of mRNA and protein expression levels of glycolysis‐related genes HK2 and PFKFB3 following UBD knockdown; (H–I) Measurement of lactate production and ATP levels in macrophages to assess glycolytic activity after UBD knockdown; (J) Detection of mRNA levels of lactate dehydrogenase LDHA after UBD knockdown using RT‐qPCR; (K) Glucose uptake assay to assess the impact of UBD knockdown on glucose uptake in macrophages. All data are presented as mean ± standard error, with all cell experiments repeated three times. iNOS, inducible nitric oxide synth; UBD, Ubiquitin D. *Indicates statistical significance between groups, ***p < 0.001, ****p < 0.0001.

First, UBD expression was silenced in RAW264.7 cells using lentivirus‐mediated gene knockdown, and the role of UBD in macrophage polarization was subsequently investigated (Figure 3A). RT‐qPCR and Western blot analysis revealed that UBD knockdown led to a significant decrease in mRNA and protein levels of the M2 macrophage marker ARG1 (Figure 3B,C). Conversely, the expression of the M1 macrophage marker iNOS was markedly upregulated, with both mRNA and protein levels of iNOS significantly elevated (Figure 3B,C). These results indicate that UBD plays a crucial role in promoting M2 macrophage polarization, and its knockdown drives a shift toward M1 polarization.

Further analysis using immunofluorescence staining confirmed these findings. After UBD knockdown, the fluorescence signal for ARG1 was notably reduced, whereas the fluorescence signal for iNOS was significantly enhanced (Figure 3D,E), providing additional evidence of UBD's role in promoting M2 polarization. These immunofluorescence results were consistent with the RT‐qPCR and Western blot findings, further supporting the critical function of UBD in regulating macrophage polarization.

Next, we assessed the impact of UBD knockdown on the expression of glycolysis‐related genes (HK2, PFKFB3). RT‐qPCR and Western blot analysis revealed that UBD knockdown significantly reduced both the mRNA and protein levels of HK2 and PFKFB3 (Figure 3F,G). These findings indicate that UBD regulates key glycolytic genes, thereby affecting macrophage metabolism. Specifically, UBD knockdown inhibits glycolytic pathways.

To further assess the effect of UBD on glycolytic metabolism in macrophages, we found that UBD knockdown significantly decreased glycolytic activity, lactate production, and ATP levels (Figure 3H,I). Meanwhile, RT‐qPCR results showed that LDHA mRNA levels were significantly reduced following UBD knockdown (Figure 3J). These data suggest that UBD knockdown suppresses glycolysis, thereby impacting the energy metabolism of macrophages.

To further confirm the effect of UBD on glycolysis, we measured glucose uptake in macrophages using a glucose uptake assay. The results showed that UBD knockdown led to a significant decrease in glucose uptake (Figure 3K). This further supports the conclusion that UBD regulates glycolytic metabolism, influencing both the energy metabolism and functional state of macrophages.

### 3.4 UBD modulates macrophage polarization to affect OC cell proliferation, migration, invasion, and immunotherapy sensitivity

**FIGURE 4:**
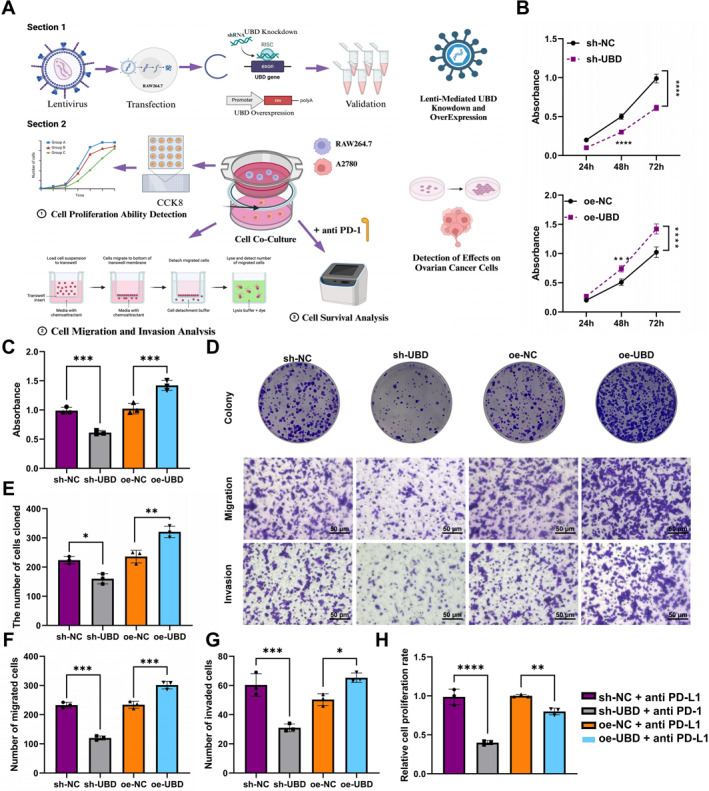
Effects of UBD Knockdown or Overexpression in Macrophages on OC Cell Proliferation, Migration, Invasion, and Immune Therapy Sensitivity. (A) Schematic illustrating the experimental procedure for evaluating the impact of UBD on macrophage regulation of OC cells; (B, C) CCK‐8 assay to assess the effect of UBD overexpression/knockdown in macrophages on OC cell proliferation; (D) Colony formation and Transwell assays to examine the effects of UBD overexpression/knockdown in macrophages on OC cell proliferation, migration, and invasion; (E) Quantitative analysis of colony formation assays; (F) Quantification of migrated OC cells co‐cultured with different groups of macrophages; (G) Quantification of invaded OC cells co‐cultured with different groups of macrophages; (H) Effect of PD‐L1 Antibody on OC cell survival in the context of UBD‐regulated macrophage activity. Data are presented as mean ± standard error, with experiments conducted in triplicate. OC, ovarian cancer; UBD, Ubiquitin D. *Indicates significance compared between groups, *p < 0.05, **p < 0.01, ***p < 0.001, ****p < 0.0001.

To evaluate the impact of UBD‐mediated glycolytic reprogramming on OC cell proliferation, we co‐cultured macrophages with UBD knockdown or overexpression with OC cells (Figure 4A). CCK‐8 assay results showed that UBD knockdown in macrophages inhibited OC cell proliferation, while UBD overexpression in macrophages promoted OC cell proliferation (Figure 4B,C). Colony formation assays confirmed that UBD knockdown in macrophages significantly suppressed OC cell proliferation, whereas UBD overexpression in macrophages significantly enhanced OC cell proliferation, consistent with the CCK‐8 assay results (Figure 4D,E).

To further explore the effect of UBD on OC cell migration and invasion, we conducted Transwell assays. The results indicated that UBD knockdown in macrophages inhibited OC cell migration and reduced the number of invading cells. In contrast, UBD overexpression in macrophages enhanced OC cell migration and invasion, with both the number of migrating and invading cells significantly increased (Figure 4D,F,G).

To investigate how UBD‐mediated glycolytic metabolic reprogramming regulates OC cell sensitivity to immunotherapy, we introduced the immune checkpoint inhibitor PD‐L1 antibody into a co‐culture system and measured OC cell viability. The results showed that in the co‐culture system with UBD knockdown macrophages, PD‐L1 antibodies inhibited OC cell survival. In contrast, in the co‐culture system with UBD‐overexpressing macrophages, the inhibitory effect of the PD‐l1 antibody was diminished (Figure 4H).

These findings suggest that UBD regulates OC cell sensitivity to immunotherapy by modulating macrophage glycolysis, and may play a key role in the development of ITR.

### 3.5 In vitro validation of UBD‐mediated macrophage polarization and its impact on OC ITR via glycolysis reprogramming

**FIGURE 5:**
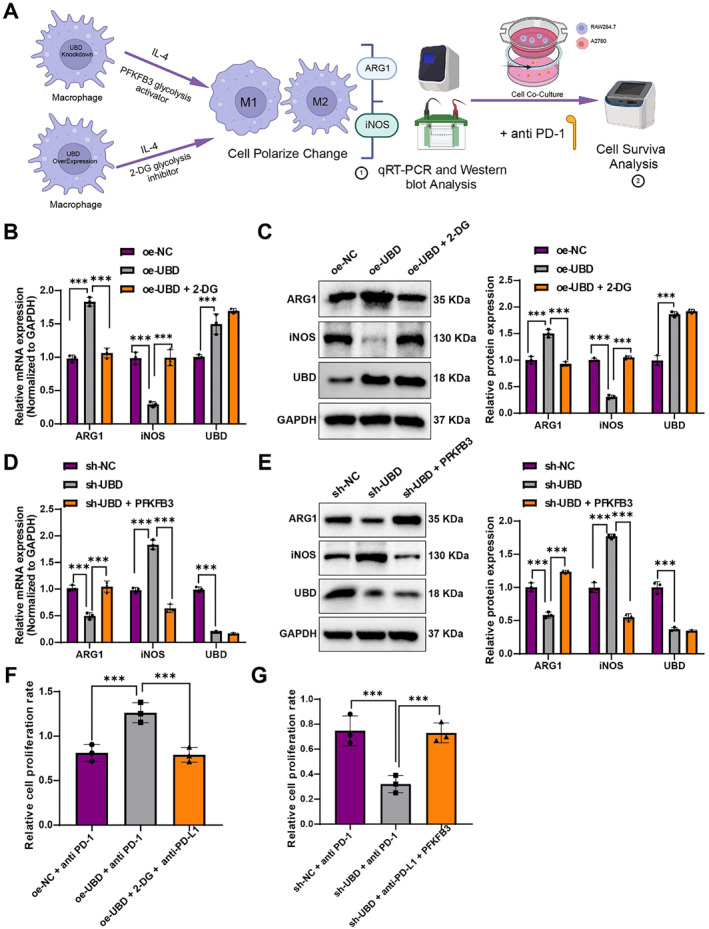
Effects of Glycolysis Inhibitor 2‐DG and Glycolysis Activator PFKFB3 on UBD‐regulated Macrophage Polarization and Sensitivity to OC Immunotherapy. (A) Experimental schematic illustrating the effect of UBD on macrophage polarization and immune response through glycolytic metabolism; (B, C) Changes in mRNA and protein levels of iNOS and ARG1 in UBD‐overexpressing macrophages after 2‐DG treatment; (D, E) Changes in mRNA and protein levels of ARG1 and iNOS in UBD‐knockdown macrophages after PFKFB3 activator treatment; (F) Effect of PD‐L1 antibody on OC cell survival in the co‐culture system of UBD‐overexpressing macrophages treated with 2‐DG; (G) Effect of PD‐L1 antibody on OC cell survival in the co‐culture system of UBD‐knockdown macrophages treated with PFKFB3 activator. All data are presented as mean ± standard error. Cell experiments were repeated three times. iNOS, inducible nitric oxide synth; OC, ovarian cancer; UBD, Ubiquitin D. * denotes comparison between groups, ***p < 0.001.

To investigate the role of glycolytic metabolic reprogramming in UBD‐mediated macrophage polarization, a series of experiments were conducted using UBD overexpression or knockdown, combined with the glycolysis inhibitor 2‐DG and the glycolysis promoter PFKFB3 activator (Figure 5A). After glycolysis was inhibited using 2‐DG, the expression of the M2 polarization marker ARG1 was increased, whereas the expression of the M1 marker iNOS was decreased in UBD‐overexpressing macrophages (Figure 5B–C). This suggests that UBD promotes M2 polarization by enhancing glycolytic pathways, and that glycolysis inhibition can reverse this effect. Conversely, when the PFKFB3 activator was applied to promote glycolysis in UBD‐knockdown macrophages, a decrease in ARG1 expression and an increase in iNOS expression were observed (Figure 5D–E).

Initially, after inhibiting glycolysis with 2‐DG, a decrease in the expression of the M2 polarization marker ARG1 and an increase in the expression of the M1 polarization marker iNOS were observed in UBD‐overexpressing macrophages (Figure 5B,C). This suggests that UBD promotes M2 polarization via glycolysis, and inhibiting glycolysis can reverse this effect. Conversely, when the PFKFB3 activator was used to enhance glycolysis in UBD‐knockdown macrophages, an upregulation of the M2 marker ARG1 and downregulation of the M1 marker iNOS were observed (Figure 5D,E). These results further confirm the critical role of glycolytic metabolism in UBD‐mediated macrophage polarization and suggest that promoting glycolysis can partially restore M2 polarization functionality in UBD‐knockdown macrophages.

To further assess the impact of these metabolic regulatory effects on OC ITR, the immune checkpoint inhibitor PD‐L1 antibody was used in co‐culture experiments, and the survival rate of OC cells was measured. The results showed that in the co‐culture system with UBD‐overexpressing macrophages and 2‐DG‐mediated glycolysis inhibition, PD‐L1 antibody significantly reduced the survival of OC cells (Figure 5F). In contrast, in the co‐culture system with PFKFB3‐activated UBD‐knockdown macrophages, the inhibitory effect of the PD‐L1 antibody was diminished (Figure 5G).

These findings demonstrate that glycolytic metabolic reprogramming, under UBD regulation, influences macrophage polarization and plays a significant role in OC ITR.

### 3.6 In vivo validation of UBD‐mediated glycolysis metabolic reprogramming regulating macrophage polarization and its impact on OC ITR

**FIGURE 6:**
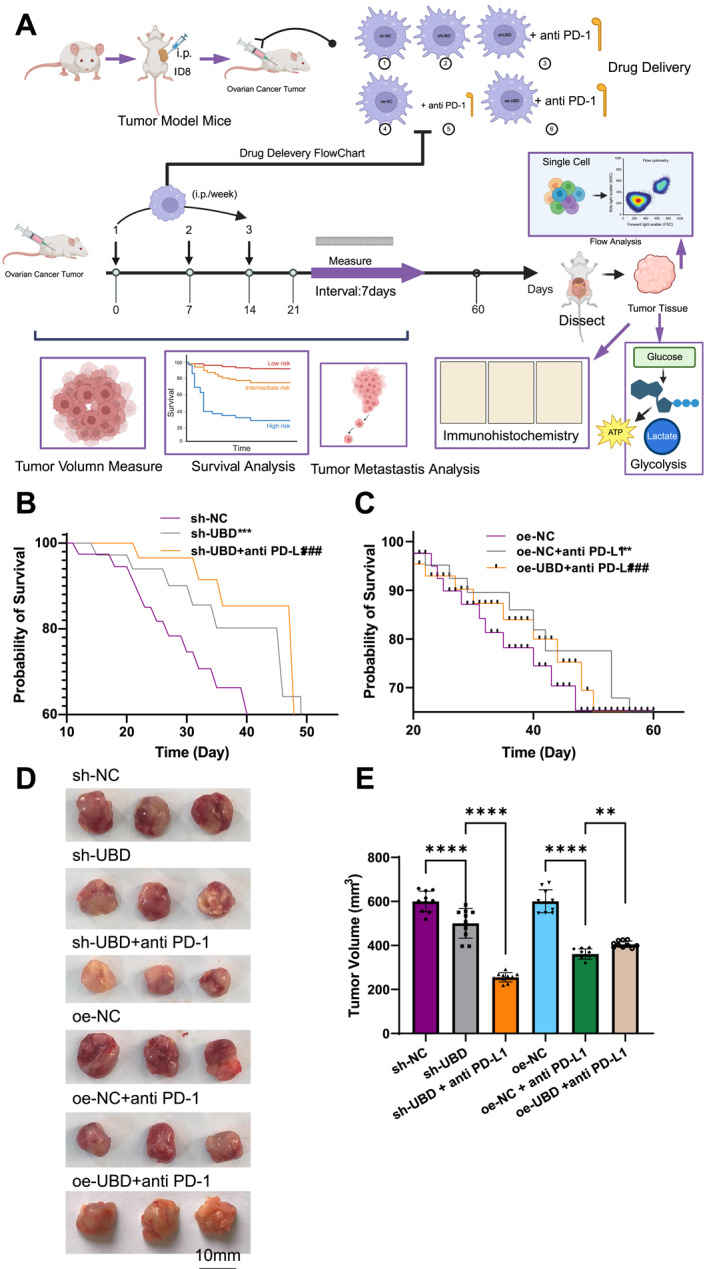
Ubiquitin D Regulates Tumor Growth and Metastasis in an ovarian cancer Mouse Model through Glycolytic Metabolic Reprogramming. (A) Schematic diagram illustrating the experimental process for assessing the effect of UBD and PD‐L1 on tumor growth in mice; (B) Kaplan‐Meier survival analysis comparing the survival time of mice in the sh‐NC and sh‐UBD groups; (C) Kaplan‐Meier survival analysis comparing the survival time of mice in the oe‐NC and oe‐UBD groups; (D) Tumor growth analysis in different experimental groups via tissue dissection; (E) Quantification of tumor volume from dissected tumors. All data are presented as mean ± standard error, with 10 mice per group in animal experiments. *Denotes comparison between groups, **p < 0.01, ****p < 0.0001.

In an OC mouse model, the experiment was divided into six groups: sh‐NC, sh‐UBD, and sh‐UBD + anti‐PD‐L1 antibody; oe‐NC, oe‐NC + anti‐PD‐L1 antibody, and oe‐UBD overexpression + anti‐PD‐L1 antibody (Figure 6A). First, we assessed the survival curves of the mice in each group. The results showed that compared to the control group, tumor growth in the sh‐UBD group was significantly slowed, and in the sh‐UBD + anti‐PD‐L1 antibody treatment group, tumor growth was further inhibited, resulting in prolonged survival (Figure 6B). In contrast, although tumor growth in the oe‐NC + anti‐PD‐L1 antibody group was moderately reduced, the therapeutic effect of anti‐PD‐L1 antibody was diminished in the oe‐UBD overexpression + anti‐PD‐L1 antibody group, and survival was not further extended (Figure 6C). Dissection of the tumor tissues revealed that the tumor volumes in the sh‐UBD and sh‐UBD + anti‐PD‐L1 antibody groups were smaller, whereas the tumor volume in the oe‐UBD overexpression + anti‐PD‐L1 antibody group was larger (Figure 6D,E). These results suggest that UBD knockdown enhances the therapeutic efficacy of PD‐L1 antibody, whereas UBD overexpression suppresses its effects, indicating that UBD, through regulating macrophage glycolysis metabolism, significantly influences OC's sensitivity to immunotherapy.

**FIGURE 7:**
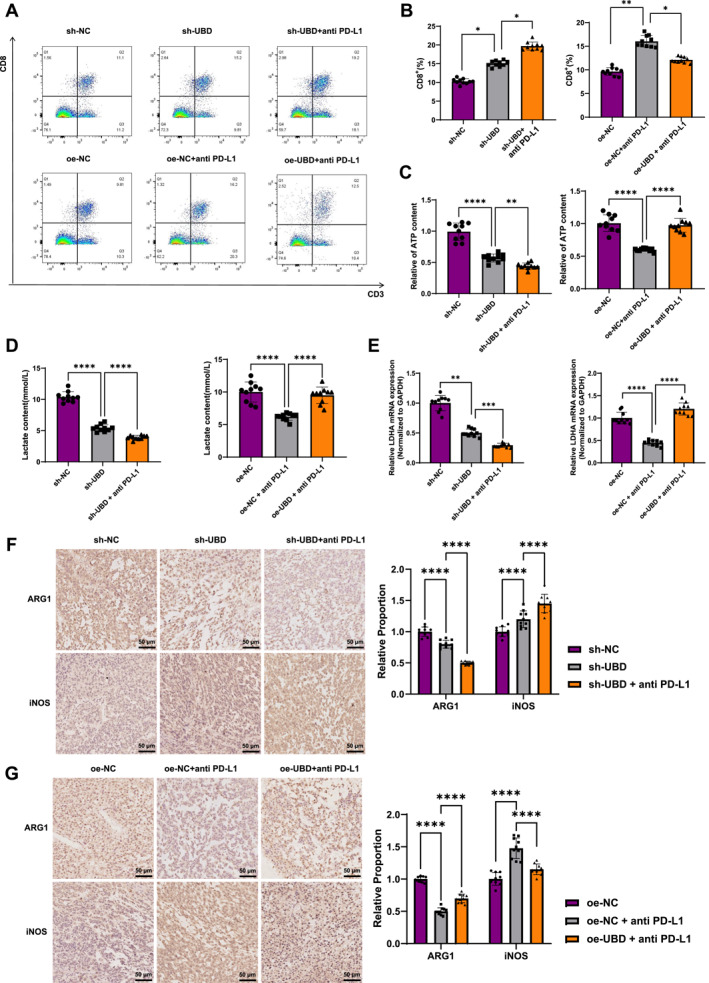
Ubiquitin D Regulates Macrophage Polarization and Tumor Immune Microenvironment via Glycolytic Metabolism. (A, B) Flow cytometric analysis of CD8^+^ T cell infiltration in tumor tissues from different treatment groups; (C, D) Colorimetric assays measuring lactate and ATP levels in tumor tissues from mice in different groups; (E) Reverse transcription quantitative polymerase chain reaction detection of mRNA level of lactate dehydrogenase LDHA in tumor tissues of different groups of mice; (F, G) Immunohistochemical detection of M2 macrophage marker ARG and M1 macrophage marker inducible nitric oxide synthase expression in tumor tissues from each group. All data are presented as mean ± standard error, with experiments repeated three times and 10 mice per group. *Indicates comparison between two groups, **p < 0.01, ***p < 0.001, ****p < 0.0001.

Flow cytometry analysis of immune cell infiltration in the tumor tissue showed that UBD knockdown increased CD8^+^ T cell infiltration, and the proportion of CD8^+^ T cells further increased after treatment with anti‐PD‐L1 antibody. In contrast, in the oe‐UBD overexpression + anti‐PD‐L1 antibody group, the proportion of CD8^+^ T cells was reduced, indicating an inhibitory effect (Figure 7A,B). These findings suggest that UBD knockdown improves the infiltration of CD8^+^ T cells in the TME, thereby enhancing the effectiveness of immunotherapy, whereas UBD overexpression diminishes this effect.

The metabolic levels of glycolysis in tumor tissues were further assessed. Upon UBD knockdown, the accumulation of lactate and ATP decreased, and their levels were further reduced in the PD‐L1 antibody treatment group. In the oe‐NC + PD‐L1 group, lactate and ATP levels were reduced, but in the oe‐UBD combined treatment group, no significant decline in lactate and ATP levels was observed (Figure 7C,D). Moreover, RT‐qPCR results indicated that LDHA mRNA expression was significantly downregulated upon UBD knockdown and further decreased with PD‐L1 antibody co‐treatment. However, in the oe‐UBD combination group, LDHA mRNA levels remained largely unchanged (Figure 7E).

Additionally, immunohistochemical analysis of macrophage polarization revealed that UBD knockdown significantly increased the proportion of M1 macrophages and decreased the proportion of M2 macrophages in tumor tissues. Following combined PD‐L1 antibody treatment, M1 polarization was further enhanced (Figure 7F). In contrast, in mice overexpressing UBD, the proportion of M2 macrophages increased while the proportion of M1 macrophages decreased, thereby inhibiting the therapeutic effects of PD‐L1 antibody (Figure 7G). These results confirm that UBD regulates macrophage polarization through glycolytic metabolic reprogramming, thereby influencing immune therapy resistance in OC.

## 4 DISCUSSION

OC is one of the most common and deadly cancers of the female reproductive system, with significant challenges in treatment, particularly regarding immune therapy resistance. In recent years, tumor cells have adapted to the TME through metabolic reprogramming, which in turn affects immune evasion and therapeutic resistance^34^, ^35^, ^36^, ^37^ UBD, an important E3 ubiquitin ligase, has been shown to play a critical role in various cancers.^20^ In this study, we found that UBD expression is significantly elevated in OC tissues and is strongly correlated with key glycolytic enzymes such as LDHA and ALDOA. Consistent with previous studies, the high expression of UBD is not only associated with tumor cell proliferation and migration but also with metabolic reprogramming in tumors. Although the role of UBD has been investigated in other cancers, such as breast and liver cancer, its specific mechanisms in OC remain poorly understood. This study systematically reveals for the first time that UBD regulates macrophage polarization through glycolytic metabolic reprogramming, providing new theoretical insights into its role in OC and suggesting that UBD may serve as a potential target for immune therapy in OC.

Macrophages are key immune cells in the TME, and their polarization directly influences tumor immune evasion and progression. M1 macrophages are pro‐inflammatory and contribute to antitumor immune responses, whereas M2 macrophages exhibit immune‐suppressive properties, facilitating tumor immune evasion.^5^, ^8^, ^9^, ^34^, ^38^ Previous studies have demonstrated that tumor cells promote growth and metastasis by modulating macrophage polarization.^39^, ^40^ Our study reveals that UBD regulates the glycolytic pathway to promote M2 polarization of macrophages and suppresses CD8^+^ T cell activity through the accumulation of lactate. Moreover, our work further shows that UBD not only influences macrophage polarization via glycolytic metabolism but also modulates metabolic products (such as ATP and lactate) to impact tumor immune evasion. Although existing literature has highlighted the relationship between metabolic pathways and macrophage polarization, the role of UBD in this process has not been systematically elucidated. Therefore, this study, through multi‐omics analysis and experimental validation, fills this research gap and provides strong evidence for a novel mechanism of UBD in macrophage polarization.

Glycolytic metabolism, known as the “Warburg effect,” refers to the phenomenon where tumor cells continue to rely on glycolysis for ATP production even in the presence of sufficient oxygen. Glycolysis plays a critical role in tumor cell proliferation and immune evasion. Recent studies have shown that metabolic byproducts, particularly lactate, in the TME can suppress immune cell function, thereby promoting tumor immune evasion.^28^, ^29^, ^41^ This study further demonstrates that UBD promotes glycolysis, leading to increased lactate production, which in turn regulates macrophage polarization and favors the formation of M2 macrophages, ultimately enhancing immune evasion in OC. This finding aligns with previous studies linking glycolysis to immune evasion. For instance, Xu et al. showed that lactate, as a metabolic byproduct, not only supports tumor cell proliferation but also inhibits T cell function. In contrast to these studies, our innovation lies in elucidating the role of glycolytic metabolism in macrophage polarization through UBD, providing a new molecular mechanism for tumor immune evasion. Although our findings show that UBD influences the expression of multiple glycolytic enzymes, the precise upstream regulatory mechanism remains to be elucidated. For example, Jiang et al. demonstrated that Zeb1 can directly regulate the transcription of glycolytic genes and modulate macrophage polarization in breast cancer,^42^ whereas Zheng et al. reported the involvement of the PDGFR‐β/TXNIP signaling axis in regulating glycolytic enzyme expression^43^ whether UBD exerts its regulatory function through these or other pathways warrants further investigation.

Immunotherapy, particularly the use of immune checkpoint inhibitors such as PD‐L1 antibodies, has become a key strategy in cancer treatment.^44^, ^45^, ^46^ However, resistance to immunotherapy remains a significant challenge, with immune evasion being one of the primary mechanisms underlying poor therapeutic outcomes. Recent studies have highlighted metabolic reprogramming as a critical mechanism driving tumor immune evasion. Our research reveals that UBD, by modulating the glycolysis metabolic pathway, influences macrophage polarization and alters the immune landscape of the TME, thereby enhancing resistance to immunotherapy. Consistent with previous studies, targeting glycolysis can improve the efficacy of immunotherapy. However, our study is unique in that it is the first to uncover the role of UBD as a metabolic regulator in tumor immune therapy. Inhibition of UBD expression significantly enhances the sensitivity of OC to PD‐L1 antibody treatment, providing strong evidence for UBD as a potential novel target for immunotherapy.

The TME is a complex milieu formed by interactions between tumor cells and surrounding immune cells, blood vessels, and stromal cells, which plays a pivotal role in regulating tumor growth and metastasis. Recent studies have shown that the metabolic state within the TME not only influences the behavior of tumor cells but also modulates immune cell functions.^47^, ^48^ In experiments conducted using an OC mouse model, we found that overexpression of UBD significantly enhanced the immunosuppressive nature of the tumor tissue, as evidenced by an increase in the proportion of M2 macrophages and a decrease in CD8^+^ T cell infiltration. Moreover, our study further confirms that UBD modulates immune cell polarization via glycolytic metabolism, thereby altering the TME and providing a novel mechanistic insight into tumor immune evasion. These findings offer a fresh perspective for exploring metabolic interventions in cancer immunotherapy.

Although this study provides new insights into the role of UBD in OC immune evasion, several limitations remain. First, the study primarily relies on cellular and mouse models. Although the experimental results are scientifically credible, their full translation into clinical practice requires further validation in clinical settings. Moreover, our current investigation focuses on the role of UBD in macrophages, particularly how it regulates M2 polarization to promote immune evasion and resistance. Whether UBD expressed in tumor cells also contributes to OC progression remains an important question that requires further exploration. Second, although we have elucidated the mechanism by which UBD regulates macrophage polarization through glycolytic metabolism, other metabolic pathways (e.g., fatty acid oxidation) have not been explored in depth. Future studies should consider the synergistic effects of multiple metabolic pathways and further investigate the comprehensive impact of UBD on tumor immune evasion.

This study is the first to demonstrate that UBD promotes M2 macrophage polarization through glycolytic reprogramming, thereby enhancing immune evasion and ITR in OC. This finding not only provides a novel theoretical basis for understanding immune evasion in OC but also offers new targeted strategies for immunotherapy. Inhibiting UBD or intervening in glycolytic pathways could be effective approaches to improve the efficacy of OC immunotherapy. Given the widespread role of UBD in other cancers, its potential application in other tumor types may provide additional avenues for precision cancer immunotherapy.

Although this study provides strong evidence for the key role of UBD in OC immune escape, further clinical research is needed to validate its specific function in patients. Future studies should focus on exploring the potential of combining UBD with other immune checkpoint inhibitors, such as CTLA‐4, to evaluate the efficacy of combination therapies. Additionally, developing novel drugs targeting UBD and its regulated metabolic pathways could offer a new direction for tumor immunotherapy. Further investigation into the downstream signaling pathways of UBD and its interactions with other metabolic pathways is crucial for a comprehensive understanding of its biological functions. Given the limited validation with clinical samples, multicenter clinical trials are necessary to assess the practical application potential of UBD. Ultimately, the development of small‐molecule inhibitors targeting UBD and exploring their applicability in other immune‐related cancers could facilitate the clinical translation of this approach.

This study systematically elucidates the mechanism by which UBD regulates macrophage polarization through glycolytic metabolic reprogramming and provides the first evidence of UBD's critical role in immune therapy resistance in OC. High UBD expression significantly promotes M2 macrophage polarization and glycolytic activity, thereby enhancing tumor immune evasion. In contrast, UBD knockdown not only reduces tumor metabolic activity but also improves CD8^+^ T cell infiltration in the TME, enhancing the therapeutic efficacy of PD‐L1 antibody treatment. These findings suggest that UBD could serve as a potential molecular target for OC therapy, and targeting UBD or intervening in glycolytic metabolism offers a novel strategy to enhance immune therapy efficacy.

## AUTHOR CONTRIBUTIONS

N.Z. and F.Z. conceived and designed the study. H.C., J.W., and H.L. performed the experiments. N.Z. and F.Z. analyzed the data. H.C., J.W., and H.L. wrote the manuscript. All authors reviewed and approved the final version of the manuscript.

## CONFLICT OF INTEREST STATEMENT

The authors declare no conflicts of interest.

## ETHICS STATEMENT

This study strictly adheres to ethical guidelines and regulations governing animal experiments. All experimental procedures were approved by the Institutional Animal Care and Use Committee (IACUC) (Ethical Approval Number: No. 120241 (005)). The animals were housed and cared for in accordance with humane principles, and efforts were made to minimize any potential pain or distress during the experiments.

## Supporting information

Table S1

## Data Availability

All data can be provided as needed.
