# Supplementary material for: UBD‐mediated glycolytic reprogramming promotes M2 macrophage polarization in ovarian cancer immune evasion
Source: J Cell Commun Signal. 2025 Jul 21;19(3):e70034. doi: 10.1002/ccs3.70034 (PMC12278697; doi:10.1002/ccs3.70034)
Supplement: Supplementary file 1 — Table S1 [file CCS3-19-e70034-s001.docx]

**Table S1.** **List of primer sequences for RT-qPCR.**

| **Primer Names** | **Sequences (5′ to 3′)** |
| --- | --- |
| ARG1 | F: ACTTAAAGAACAAGAGTGTGATGTG |
|  | R: ATTGCCAAACTGTGGTCTCC |
| iNOS(NOS2) | F: AACAGGGAGAAAGCGCAAAA |
|  | R: CCTCACATACTGTGGACGGG |
| PFKFB3 | F: AGAGGTCAGAGAACATGAAGAGC |
|  | R: ACATGCCGACCTCCATTCTC |
| HK2 | F: CTGCTTTGGAGATCCGAGGG |
|  | R: GTCTAGCTGCTTAGCGTCCC |
| LDHA | F: CCAATATGGCAACTCTAAAGGATC |
|  | R: GCAAGTTCATCTGCCAAGTCCT |
| GAPDH | F: AAGCCCATCACCATCTTCCAGGAG |
|  | R: AGCCCTTCCACAATGCCAAAG |

Note: F: forward; R: reverse.
